# Chyloperitoneum After Penetrating Abdominal Trauma: A Report of a Rare Case

**DOI:** 10.7759/cureus.40188

**Published:** 2023-06-09

**Authors:** Georgia Syrnioti, Ghayth Al Awwa, Jacob Eisdorfer

**Affiliations:** 1 Surgery, Brookdale University Hospital Medical Center, New York, USA

**Keywords:** total parenteral nutrition (tpn), medium-chain fatty acids, octreotide, penetrating abdominal injury, chylous ascites, chyloperitoneum

## Abstract

The term chyloperitoneum refers to the accumulation of triglyceride-rich fluid in the peritoneal cavity. It is an uncommon clinical condition that usually occurs due to disruption of lymphatic flow secondary to trauma or obstruction. Common causes include penetrating or blunt trauma, iatrogenic injuries, congenital anomalies, malignant neoplasms, infections such as tuberculosis and filariasis, liver cirrhosis, constrictive pericarditis, congestive heart failure, inflammatory conditions, such as sarcoidosis and pancreatitis, and radiation- and drug-related pathologies. We present a case of chyloperitoneum in a 33-year-old woman secondary to penetrating abdominal trauma secondary to a gunshot wound. The patient was successfully managed with total parenteral nutrition and octreotide administration. To our knowledge, this is the only case of chylous ascites caused by a penetrating injury that has been reported in the literature. Conservative management with the initiation of total parenteral nutrition and octreotide led to the resolution of this condition.

## Introduction

The term chyloperitoneum refers to the accumulation of triglyceride-rich fluid in the peritoneal cavity. It is an uncommon clinical condition that usually occurs due to disruption of lymphatic flow secondary to trauma or obstruction [1].

The lymphatic system is a network of tissues that allows the return of excess interstitial fluid to the vasculature. In the intestine, the major function of gut lymphatics is to regulate the interstitial fluid balance and transportation of lipids [2]. Lymph from the gastrointestinal and lumbar region drains into the cisterna chyli, an area of saccular dilatation that is formed at the level of the first and second lumbar vertebral body, and lies laterally to the right crus of the diaphragm behind the abdominal aorta [3].

Disruption of the flow of the lymph regardless of the etiology can result in extravasation of the lymph that can manifest itself as chylous ascites. Common causes include penetrating or blunt trauma, iatrogenic injuries, congenital anomalies, malignant neoplasms, infections such as tuberculosis and filariasis, liver cirrhosis, constrictive pericarditis, congestive heart failure, inflammatory conditions, such as sarcoidosis and pancreatitis, and radiation- and drug-related pathologies [1]. Chylous ascites can result in the depletion of nutrients, electrolytes, and immunoglobulins, and therefore prompt diagnosis and management are necessary [2].

This article presents a case of chyloperitoneum secondary to penetrating abdominal trauma due to a gunshot wound. To the authors’ knowledge, this is the only case of chylous ascites caused by a penetrating injury that has been reported in the literature.

## Case presentation

**Figure 1:**
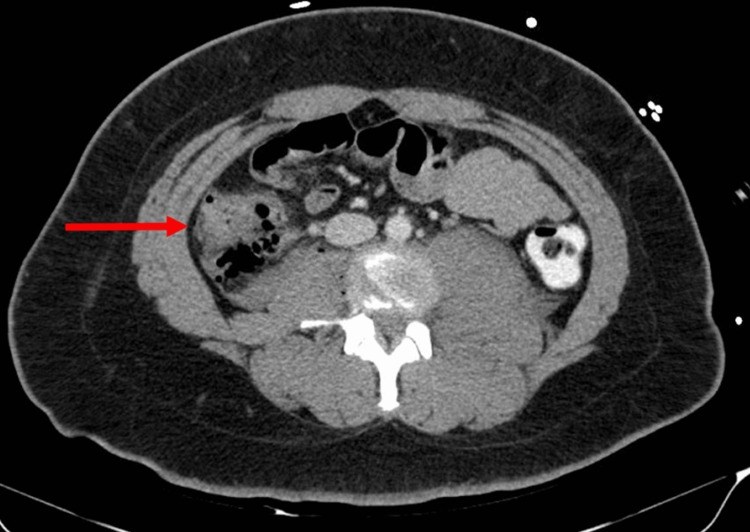
Presence of extraluminal air in the right paracolic gutter (red arrow) suspicious for right colonic injury.

**Figure 2:**
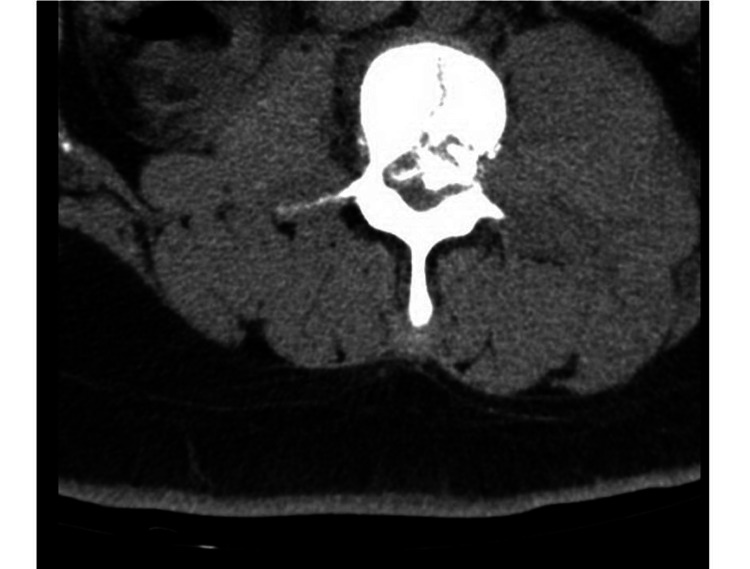
Acute comminuted fracture of the L3 vertebral body with retropulsion and compression of the spinal canal.

A 33-year-old female with no past medical and surgical history of sleeve gastrectomy was brought to the emergency department after sustaining a gunshot wound to the left flank. The patient was hemodynamically stable upon arrival. Bilateral lower extremity weakness with motor strength 3/5 was noticed. Focused Assessment with Sonography for Trauma (FAST) was negative. Computed tomography (CT) chest/abdomen/pelvis with intravenous contrast and CT of the thoracic and lumbar spine were performed, which revealed evidence of right colonic injury with extraluminal air and fluid in the right paracolic gutter along with an acute comminuted fracture of the L3 and L4 vertebral bodies with retropulsion and compression of the spinal canal (Figures 1, 2).

The patient was taken to the operating room for exploratory laparotomy. Intraoperative findings included a trans-retroperitoneal bullet trajectory from the left flank, left psoas, and L3/4 vertebral bodies through the right psoas below the inferior renal pole and ascending colon with gross contamination. Right hemicolectomy with primary anastomosis and abdominal washout were performed.

**Figure 3:**
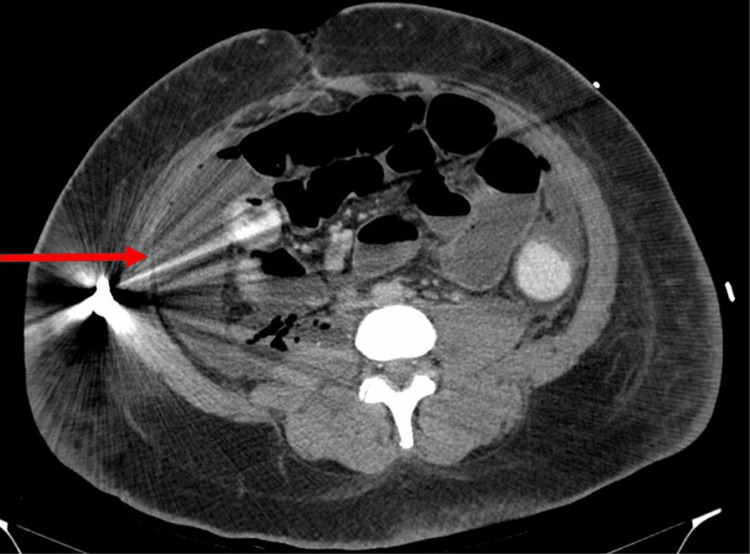
Presence of extraluminal fluid and gas tracking into the right retroperitoneum along the anterior psoas muscle margin. The fluid collection measures 7.4 x 2.4 cm (red arrow).

**Figure 4:**
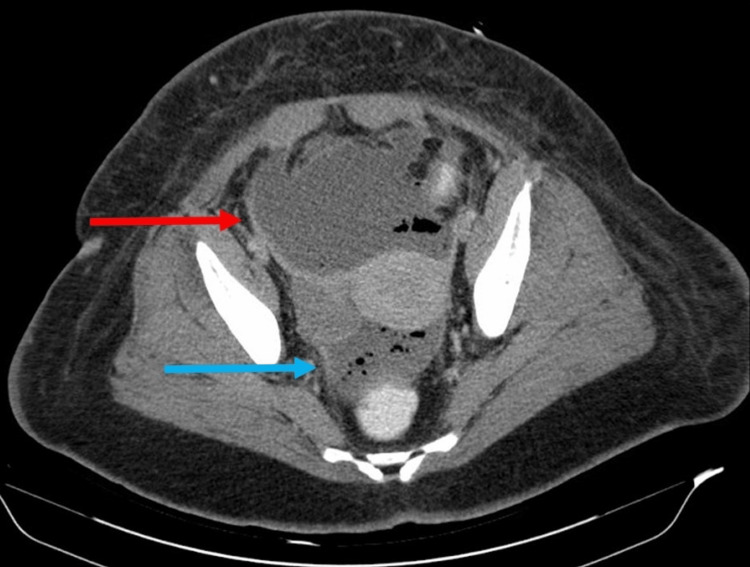
Presence of large fluid collections, one anterior to the uterus measuring 10 x 7.2 cm (red arrow) and the other between the uterus and the rectum (blue arrow) measuring 6.5 x 3.8 cm.

The patient had a prolonged hospitalization that was complicated by the presence of intra-abdominal collections that were initially identified on postoperative day 7 (Figures 3, 4).

Paracentesis of the abdominal collections with the placement of a pigtail catheter was performed twice by interventional radiology.

The patient was noted to have an ongoing increased output of milky, odorless fluid from the drains (approximately 200-400 mL daily) with eventual dehiscence of the midline abdominal incision.

**Figure 5:**
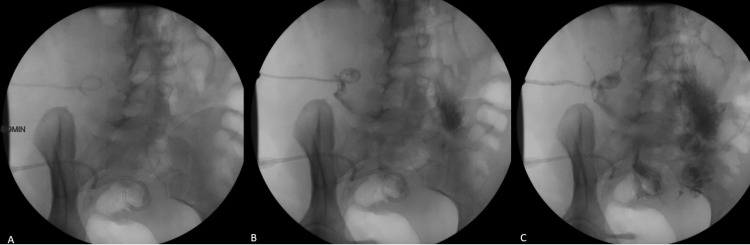
After injection of water-soluble contrast into the 8 Fr drain, there is outlining of loops of the bowel with no evidence of intraluminal contrast suggesting a connection to the bowel. Deeper within the pelvis, there is outlining of colonic haustra but no evidence of passage of contrast into the lumen of the bowel.

Differential diagnosis included a missed renal/ureteral injury, the presence of a pancreatic fistula, and the very rare case of chylous ascites secondary to disruption of the cisterna chyli that lies adjacent to the first and second lumbar vertebral bodies. Although the collection was initially presumed to be infectious in etiology, this diagnosis was excluded given the absence of systemic findings, benign abdominal examination, diet tolerance, and failure to respond to antibiotics. A CT abdomen/pelvis with enteric contrast did not reveal any communication between the collections and the gastrointestinal tract. CT cystogram and ureterogram were performed, which did not show any evidence of a urinary leak. Indigo carmine dye was also administered through the urinary catheter in an attempt to highlight the drain output in case there was communication with the urinary system, but the test was negative. A fistulogram was also performed with the administration of water-soluble contrast through one of the pigtail catheters; however, no communication was identified between the collections and the gastrointestinal/ urinary systems (Figure 5).

The diagnosis of pancreatic fistula was rather unlikely due to the presence of low drain amylase (10 U/L). Given the high suspicion of chyloperitoneum, lymphoscintigram was performed, but unfortunately the study was limited and non-diagnostic. Triglycerides from the drains were sent and were initially within normal limits (30 mg/dL and 43 mg/dL, normal values <65 mg/dL). The patient was given a fatty meal, and the exam was repeated. Drain triglycerides were 90 mg/dL. Given the high suspicion of chyloperitoneum, total parenteral nutrition (TPN) and octreotide 100 mcg three times daily were initiated and administered for one month. She was then initiated on a low-fat diet with medium-chain fatty acids and tolerated it well. The patient demonstrated significant improvement of the midline abdominal wound dehiscence, while the drain output gradually decreased. Drains were removed on postoperative day 66. The patient was discharged after 72 days of hospitalization. Complete closure of the abdominal wound dehiscence was evident three months after discharge. 

## Discussion

The term chyloperitoneum refers to the accumulation of triglyceride-rich, milky-appearing fluid in the abdominal cavity. It is an uncommon clinical entity that was first described by Morton in 1694 as a result of disruption of the cisterna chyli in the setting of tuberculosis [4]. The prevalence of chyloperitoneum was approximately one in 20,000 admissions at a large university-based hospital over a two-decade period as reported by Press et al. [5]. Since no further epidemiologic studies have been conducted, the exact prevalence is unknown. However, it is speculated that the incidence has increased due to prolonged survival of cancer patients and aggressive surgical interventions.

The majority of cases are due to malignancies (predominantly lymphoma), cirrhosis, and iatrogenic mechanisms/injuries due to disruption of the lymphatic flow during complex general surgery cases. In the pediatric population, the etiology is most likely congenital [1]. Traumatic chyloperitoneum is a rare entity and is usually caused by blunt abdominal trauma that results in tears of the root of the small bowel mesentery [6]. Due to the high intensity of the trauma, it is usually associated with other injuries, although isolated cases have also been reported [7].

The clinical picture of a patient developing chylous ascites is usually insidious with gradual accumulation of peritoneal fluid and increased abdominal girth. As the disease progresses, vague abdominal pain, nausea, and paralytic ileus may appear [1]. In our case, the presence of excess chylous fluid in the peritoneal cavity led to dehiscence of the midline abdominal incision and leakage of milky-appearing fluid. Prolonged loss of chyle in the third space can lead to dehydration, immunosuppression, and electrolyte imbalances [6].

Several diagnostic tools are available. CT of the abdomen is particularly useful in identifying intra-abdominal collections. In the setting of chyloperitoneum, the accumulated fluid has a similar density to water and is readily distinguishable from hemorrhagic fluid, especially in the context of trauma [1]. The pathognomonic feature of chyloperitoneum in CT abdomen is the presence of a fat-fluid level, which can be demonstrated after placing the patient in the supine position for a prolonged time. Because lipids have a specific gravity that is lower than water, a fat-fluid level can be formed. This sign can also be visible with abdominal ultrasonography [8].

Lymphoscintigraphy can also be used in order to demonstrate obstruction or leakage of lymphatic channels. It is performed with subcutaneously injected technetium-99m-labeled colloid, which, due to its high specificity for the lymphatic system, can provide useful information about the transport of the lymph. In the presence of a leak fluid, collections can be identified due to the high uptake of the radionuclide tracer. The limitation of the study arises from low spatial resolution and lack of spatial information [8]. The use of the hybrid single-photon emission computed tomography (SPECT)/CT can provide morphologic and anatomic separation and is especially useful when surgical intervention is planned.

Lymphangiography is considered to be the gold standard in defining chylous ascites secondary to obstruction [8]. Besides its diagnostic value, lymphangiography also has a potential therapeutic role in the management of chylous ascites through the induction of inflammatory reactions in the lymphatic vessels and subsequent embolization of the chylous leak [9].

Abdominal paracentesis is the most important confirmatory test [1]. Chyle typically has a milky white, cloudy, and turbid appearance. The fluid is characterized by an elevated triglyceride concentration (often >1000 mg/dL), lymphocyte predominance, and elevated total protein and cholesterol concentrations [8].

The management of chylous ascites is usually conservative and aims to limit the chyle flow through the intestinal lymphatics. Dietary restrictions consist of the administration of a high-protein, low-fat diet with medium-chain triglycerides (MCT) [1, 6]. In contrast to long-chain triglycerides (LCT) that are absorbed through gut lymphatics, MCTs are absorbed directly into the intestinal cells and are transported to the liver via portal circulation. In refractory cases, TPN should be implemented [1]. Although it decreases the production and flow of lymph, TPN is associated with prolonged length of stay, as was evident in our patient, is expensive, and can be complicated by bloodstream infections [6].

Medical therapy consists of several different medications that aim to decrease the accumulation of chylous fluid in the peritoneal cavity. Somatostatin and its synthetic analog octreotide have been used in the management of chylous ascites. Although their mechanism is not completely understood, they have been shown to decrease portal venous pressure by inhibiting splanchnic vasodilation. They can also decrease intestinal peristalsis, fat absorption, thoracic duct flow, and triglyceride content in lymphatics [10]. Somatostatin has a half-life of one to three minutes and is administered intravenously, while octreotide has a half-life of two hours and is given subcutaneously [11]. Orlistat, a reversible inhibitor of gastric and pancreatic lipases, has been used in the management of chylous ascites secondary to cirrhosis; however, no data exist on its efficacy on traumatic chyloperitoneum [12]. Etilefrine, an alpha-1 adrenergic agonist that is used in postural hypotension, has also been reported to be used in the management of chylous ascites after pancreatectomy and esophagectomy [13]. Through its sympathomimetic action, etilefrine induces contraction of the thoracic duct, leading to reduced flow of chyle. Etilefrine is currently not available in the United States.

If the chylous ascites does not respond to conservative measures, surgical management should be pursued. Abdominal paracentesis and drainage of the abdominal fluid play both diagnostic and therapeutic roles. Paracentesis should be used in combination with other conservative measures due to its decreased efficacy when used alone [14]. Lymphagiography and embolization have also been used in the management of chylous ascites. Lymphatic embolization has been reported to be therapeutic in 56-86% of patients [15, 16]. Complications of embolization are rare and are usually the result of the migration of the glue into the systemic venous system through lymphaticovenous anastomoses [17].

Peritoneovenous shunting (LeVeen or Denver shunts) has been used in the past in the management of refractory cases, but their use is currently limited due to the high rate of shunt occlusion, sepsis, electrolyte imbalance, small bowel obstruction, and disseminated intravascular coagulation [18]. Surgical repair is the most direct solution; however, the timing remains unclear and the decision should be individualized. A lymphoscintigraphy or lymphangiography can be used preoperatively in order to identify the source of the leak [19]. In these cases, open surgical ligation of the lymphatic leak or the implantation of a peritoneovenous shunt is an option [20].

## Conclusions

Chyloperitoneum is a rare cause of ascites after traumatic injury. Although cases of chylous ascites have been described in the setting of blunt trauma, it can also be caused secondary to penetrating abdominal injury. For this reason, chylous ascites should be included in the differential diagnosis of intra-abdominal collections after abdominal trauma. The diagnostic and therapeutic management of traumatic chyloperitoneum remains a dilemma. Conservative management should be the first choice; however, surgical repair may be pursued in refractory cases.
